# Diagnostics and treatment of impulse control disorders, psychosis and delirium: systemic review-based recommendations - guideline “Parkinson’s disease” of the German Society of Neurology

**DOI:** 10.1007/s00415-024-12576-x

**Published:** 2024-07-24

**Authors:** Karsten Witt, Johannes Levin, Thilo van Eimeren, Alkomiet Hasan, Georg Ebersbach, for the German Parkinson’s Guideline Group, Mathias Bähr, Jos Becktepe, Daniela Berg, Kathrin Brockmann, Carsten Buhmann, Andrés Ceballos-Baumann, Joseph Claßen, Cornelius Deuschl, Günther Deuschl, Richard Dodel, Georg Ebersbach, Carsten Eggers, Thilo van Eimeren, Alessandra Fanciulli, Bruno Fimm, Ann-Kristin Folkerts, Madeleine Gausepohl, Alkomiet Hasan, Wiebke Hermann, Rüdiger Hilker-Roggendorf, Günter Höglinger, Matthias Höllerhage, Franziska Hopfner, Wolfgang Jost, Elke Kalbe, Jan Kassubek, Stephan Klebe, Christine Klein, Martin Klietz, Thomas Köglsperger, Andrea Kühn, Paul Krack, Florian Krismer, Gregor Kuhlenbäumer, Johannes Levin, Inga Liepelt-Scarfone, Paul Lingor, Kai Loewenbrück, Matthias Löhle, Stefan Lorenzl, Sylvia Maaß, Walter Maetzler, Regina Menzel, Philipp T. Meyer, Brit Mollenhauer, Manuela Neumann, Per Odin, Tiago Outeiro, Monika Pötter-Nerger, René Reese, Kathrin Reetz, Olaf Rieß, Viktoria Ruf, Anja Schneider, Christoph Schrader, Alfons Schnitzler, Klaus Seppi, Friederike Sixel-Döring, Alexander Storch, Lars Tönges, Claudia Trenkwalder, Thilo van Eimeren, Uwe Walter, Tobias Wächter, Tobias Warnecke, Florian Wegner, Christian Winkler, Karsten Witt, Dirk Woitalla, Kirsten Zeuner, Martina Bantel, Jonas L. Witt

**Affiliations:** 1 Department of Neurology, School of Medicine and Health Sciences, Carl von Ossietzky University of Oldenburg, Marienstrasse 15, 26121 Oldenburg, Germany; 2 University Clinic of Neurology, Evangelical Hospital, Oldenburg, Germany; 3 Center of Neurosensory Sciences, University of Oldenburg, Oldenburg, Germany; 4 Department of Neurology, LMU University Hospital, LMU Munich, Munich, Germany; 5 German Center for Neurodegenerative Diseases, Site Munich, Munich, Germany; 6 Munich Cluster for Systems Neurology (SyNergy), Munich, Germany; 7 Department of Neurology, University of Cologne, Cologne, Germany; 8 Department of Psychiatry, Psychotherapy and Psychosomatics, Faculty of Medicine, University of Augsburg, Augsburg, Germany; 9 DZPG (German Center for Mental Health), Partner Site München/Augsburg, Augsburg, Germany; 10 Movement Disorder Hospital, Beelitz-Heilstätten, Germany

**Keywords:** Parkinson’s disease, Impulse control disorder, Psychosis, Delirium, Guideline

## Abstract

**Background and objective:**

Impulse control disorders (ICD), psychosis and delirium are part of the spectrum of behavioural changes associated with Parkinson’s disease (PD). The diagnostic and therapeutic management of these rather complex neuropsychiatric conditions has been updated in the clinical guideline by the German Society of Neurology (DGN).

**Methods:**

Recommendations are based on a systematic literature reviews, other relevant guidelines and expert opinion.

**Results:**

Patients receiving dopamine agonists (DA) therapy should be informed about the symptoms and risks of an ICD and should be routinely screened for ICD symptoms. In the presence of an ICD, DA should be reduced or discontinued and psychotherapeutic treatment may be considered. Non-oral therapies (levodopa/carbidopa intestinal gel infusion or deep brain stimulation) may also be an option for appropriate candidates. Psychosis in PD often has a gradual onset. Cognitive and affective disorders, psychiatric and medical comorbidities as well as polypharmacy are risk factors for a psychosis. Non-pharmacological treatments should be implemented as soon as possible and anti-parkinsonian medications should be adjusted/reduced if feasible. For psychosis associated with PD, quetiapine or clozapine should be used on an as-needed basis and for as short a time as is necessary, with safety monitoring. Delirium in PD may be underdiagnosed due to an overlap with chronic neuropsychiatric features of PD. Although transient by definition, delirium in PD can lead to permanent cognitive decline, motor impairment and increased mortality. Management of delirium includes pharmacological and non-pharmacological interventions.

**Conclusion:**

The updated guideline encompasses the evidence-based diagnostic, non-pharmacological and pharmacological management of ICD, psychosis and delirium in PD.

## Introduction

Parkinson’s disease (PD) is a neuropsychiatric disorder [1]. In addition to the motor symptoms, the spectrum for PD symptoms includes a wide range of neuropsychiatric manifestations. A meta-analysis of 30 studies including 7142 PD patients with a disease duration of more than 3 years reported a prevalence of depressive disorders of 47.2%, apathy of 45.5%, anxiety disorders of 42.9%, psychotic disorders of 19.4% and impulse control disorders (ICD) of 18.5% [1]. A prevalence of 31% has been reported for delirium in inpatients with PD [2]. The German 2016 guideline included the medical management of psychosis in PD [3]. The new guideline from 2023 updates the diagnostic and therapeutic management of psychosis in PD and newly includes the diagnosis and treatment of ICD and delirium in [4]. In the present article, the specified diagnostic and therapeutic management issues, results of a systemic literature review, other relevant guidelines and expert opinions are used to generate recommendations for these disorders in PD based on the recently published German guideline.

### What is new in the 2023 guideline?

In addition to the previous version, the new guidelines include recommendations for the diagnosis and treatment of ICD, which are important given the high prevalence in PD. Risk factors, diagnosis and treatment of psychosis in PD have been updated and the recommendations for the diagnosis and management of delirium are included. Main recommendations are as follows:

*Impulse control disorders*

- Before using dopamine agonists, patients should be informed about the signs and symptoms of an ICD and their association with the use of dopamine agonists.
- The Questionnaire for Impulsive-Compulsive Disorders in Parkinson's Disease" (QUIP) should be used to assess signs and symptoms of ICD.
- The QUIP test as a screening test should be aided by patient’s history and expand with an external medical history.
- Stopping dopamine agonists is an effective ICD treatment. We recommend a gradual doses reduction of dopamine agonists.
- In the event of adverse effects of dopamine agonist reduction (e.g. dopamine agonist withdrawal syndrome), the dopamine agonist should be reduced to the lowest tolerated dosage.
- Cognitive behavioural therapy can be used to treat ICD in PD**.**
- Non-dopaminergic drugs such as naltrexone, zonisamide, clozapine, risperidone, quetiapine and valproate acid should not be used for ICD therapy given the lack of evidence.
- PD patients eligible for a continuous non-oral dopaminergic therapy, levodopa-carbidopa intestinal gel (LCIG) therapy may be considered for ICD therapy.
- Bilateral deep brain stimulation of the subthalamic nucleus can be recommended to treat signs and symptoms of an ICD in patients eligible for deep brain stimulation.

*Psychosis*

- A stepwise approach is recommended in PD patients with psychotic symptoms:
- Implementation of general non-pharmacological measures (e.g. stimulus shielding, improvement of sensory impressions by glasses or hearing aid supply, reorientation measures, restoration of circadian rhythm)
- Implementation of general therapeutic measures (including treatment of an dehydration or infection)
- Reduction/adjustment of triggering drugs in general (anticholinergic, antiglutamatergic, sedative) and anti-parkinsonian drugs (especially amantadine, MAO-B inhibitors, dopamine agonists and COMT inhibitors, combination treatments).
- If 1–3 fail, clozapine should be used in the balance between benefits and risks (risk of agranulocytosis, risk of myocarditis, risk of falls, anticholinergic side effects). Alternatively, quetiapine may be offered off-label in PD patients without cognitive impairment.
- In the case of cognitive impairment and failure of 1–3, a switch to an acetylcholinesterase inhibitor can be offered.
- If 1–4 and possibly 5 fail, electroconvulsive (ECT) treatment can be offered.

*Delirium*

- Amantadine, anticholinergics and dopamine agonists should be reduced or (gradually) discontinued.
- Non-pharmacological measures for prevention and therapy of delirium in PD include early mobilisation, avoidance of catheters and accesses, adherence to the day–night rhythm, sensory aids (hearing aids, visual aids), food and fluid intake, quiet environment with good lighting and good colour contrasts, treatment of pain and infections, presence of caregivers (including rooming-in) and reorientation measures (e.g. introduction by name at every contact, clock in the field of vision)
- In the case of delirium in PD, triggering factors (pain, infections, metabolic disorders) should be treated with medication.
- Quetiapine may be used to treat delirium. When using quetiapine, cognition, blood pressure and bladder emptying should be monitored. Clozapine and benzodiazepines may be used in individual cases with special consideration of possible side effects, especially the risk of falls.

## Methods

The new guidelines were developed as S2k guideline, i.e. the recommendations were based on a structured consensus process.

The guidelines follow the rules and recommendations of the Sk2 guidelines of the AWMF (Arbeitsgemeinschaft der Wissenschaftlichen Medizinischen Fachgesellschaften/Working Group of the Scientific Medical Societies) [5]. The writing team for the chapters on ICD, psychosis and delirium consisted of an interdisciplinary group of experts from the fields of neurology (KW, JL, TvE, GE) and psychiatry and psychotherapy (AH). The key questions selected for the development of the recommendations were developed by this team and approved by the Editorial Board of the PD Guidelines, which consists of the publisher, i.e. the German Neurological Society (DGN), other professional associations and organisations, and experts in the field. The authors of this guideline developed a draft guideline, which was discussed and voted on as part of a structured consensus building process by a panel of experts (see appendix) and 22 German, Austrian and Swiss professional societies and organisations, including patient representatives.

The recommendations are based on (i) systematic reviews in the MEDLINE database, including meta-analyses, randomised controlled trials (RCTs) and other relevant studies; (ii) German national guidelines for delirium [6] and schizophrenia [7]; (iii) recommendations from the International Parkinson and Movement Disorder Society (MDS); (iv) expert opinion based on clinical expertise and knowledge of the literature and (v) the previous version of the Parkinson’s Disease (PD) guideline [8] for all questions that were already included.

The recommendations follow a “should/should not”, “can/cannot” or “might/might not” sentence structure. The recommendations were discussed and approved by the PD Guidelines Editorial Board. Revisions were made based on the discussions. The final recommendations were then voted on and scored according to agreement (strong agreement: > 95% positive votes; agreement: > 85–95%; majority agreement: > 50–85%; no majority agreement: < 50%).

## Results

### Impulse control disorders

Impulse control disorders (ICD) are defined as a “failure to resist an impulse, temptation (impulsivity), or drive to perform an act (compulsivity) that is harmful to the person or others (functional impact)” [9]. In the largest study to date to survey ICD symptoms in over 3,000 PD patients, ICD was found in 14% of patients, a third of whom suffered from multiple symptoms of ICD [10]. A multicentre French study showed a 5-year incidence of 46% for ICD in PD patients taking dopamine agonists (DAs) [11]. ICD consists mainly of four behavioural disorders:

- Gambling addiction is defined as persistent or repeated gambling behaviour that harms the patient through loss of money. Gambling cessation is associated with dysphoria. The gaming behaviour is uncontrollable for patients [12].
- Pathological buying behaviour is a persistent or repeated buying behaviour that is out of proportion to the buyer's income or for which there is no benefit. It often results in financial loss and debt [13].
- Hypersexuality is defined by an abnormal interest or excessive preoccupation with sexual content, thoughts and pornography and may be associated with inappropriate self-stimulatory behaviour or use of sexual services. It is rarely associated with exhibitionistic behaviour, paraphilia or zoophilia [14].
- Binge eating is defined as inappropriately high food intake over a short period of time that leads to weight gain and can be associated with damage to the body’s metabolism [15].

Although the problems mentioned here reflect the majority of ICD, very individual pathological behaviours with the characteristics of ICD may be seen in individual cases, including tattooing, kleptomania, reckless driving and many other areas. Other behavioural abnormalities can be distinguished from ICD, such as dopaminergic dysregulation syndrome (DDS) and punding [16]. DDS is characterised by inappropriate use of dopaminergic drugs, usually fast-acting drugs such as soluble levodopa, beyond the dose that would be sufficient to control motor symptoms [17]. Punding is repetitive, unproductive and often stereotyped behaviour [16].

### Which tests are suitable for the assessment of impulse control disorders in patients with PD compared to PD patients without impulse control disorders?

The wide clinical spectrum of behavioural abnormalities in PD requires a detailed history in addition to the use of a standardised tests. This is because ICD are not necessarily associated with personal distress for the patient, and there are even feelings of satisfaction when ICD behaviours are performed [11]. Therefore, an external history is recommended. As ICD can occur at any time during the illness and also with a marked latency to the initiation of DA treatment [18], sustained attention is required to detect ICD in PD. As ICD is associated with self-harm and harm to others and significantly reduces the patient's quality of life [19], appropriate diagnosis is essential.

### Data sources

The literature search identified 386 publications. After screening the abstracts and suitable full texts, no further studies were extracted from the literature references.

### Study analysis and guideline establishment

The Questionnaire for Impulsive-Compulsive Disorders in Parkinson's disease (QUIP) is available as a validated translation into German [20, 21]. The scale has three sections. The first section asks about signs and symptoms of an ICD (gambling, shopping, hypersexuality and eating disorders). Section 2 asks about other compulsive behaviours (punding, hobbyism and ritualistic walking) and section 3 asks about medication overuse. The QUIP is used to assess the presence of ICD symptoms both during the course of the illness (QUIP-anytime) and at the time of the interview (QUIP-current). The questionnaire is completed using the QUIP Rating Scale (QUIP-RS), which describes the severity of symptoms [20]. The German version of the QUIP shows a good sensitivity for ICD symptoms of 68–91% for the QUIP-current and 68–91% for the QUIP-anytime [21]. Whenever the QUIP indicates to the presence of an ICD a detailed psychiatric examination should be performed in order to assess the individual extent and severity of the symptoms.

DA use increases the risk of ICD by a factor of 2–3.5 and is therefore the most important risk factor for an ICD in PD. Patient-related risk factors include a family or personal history of alcohol or gambling addiction, younger age, male sex, depression, anxiety disorders, early age of disease onset [10] and the presence of alexithymia [3]. Other risk factors are of genetic origin, including polymorphisms in the dopaminergic system (DRD1, DRD2, DRD3, DRD4, COMT, DAT) [22] and are not indicated for routine clinical testing. Identification of risk factors for ICD in PD can stratify an individual’s risk profile, which may be useful in terms of patients’ education, likelihood of developing an ICD and frequency of screening for ICD symptoms.

**Table:** 

*Recommendation for IDC symptom screening (new)*
Before using dopamine agonists, patients should be informed about the signs and symptoms of an ICD and their association with the use of dopamine agonists The Questionnaire for Impulsive-Compulsive Disorders in Parkinson’s Disease (QUIP) should be used to assess signs and symptoms of impulse control disorders The QUIP test as a screening test should be aided by a patient history and an external medical history
Level of consensus: 96.6%, strong consensus

### How effective is a change in dopaminergic drug therapy for Parkinson's disease in the treatment of impulse control disorders in PD compared to no change in drug therapy?

DA use increases the risk of ICD by a factor of 2–3.5. The pathophysiology of ICD is incompletely understood and includes (i) impaired mesolimbic dopaminergic function (reward prediction error) following drug-induced postsynaptic stimulation [23, 24], (ii) impaired inhibitory control [25, 26] and (iii) impaired top-down control of reward evaluation [27–29]. Although in this model DA use is the main driving factor for an ICD, ICDs have also been described with higher doses of levodopa [10], amantadine [30], levodopa/carbidopa intestinal gel [31] and MAO-B inhibitors [32], which may be explained by their direct or indirect dopaminergic action.

ICD has been described with all non-ergot DAs [32–34]. The individual DAs are associated with a different risk of developing an ICD, with pramipexole and ropinirole having a higher risk of ICD than the DA rotigotine [33], which may be explained by differences in DA pharmacokinetics and pharmacodynamics.

### Data sources and search strategy

A literature search in PubMed identified 532 publications, which were analysed for the three sub-questions (see below) using title, abstract and, where appropriate, full text.

The key question can be divided into three subdivisions, which are reported here:

1. The efficiency of switching between DAs for ICD therapy. Retrospective studies and case series were identified. Randomised studies are not available.
2. The efficiency of reducing the dose of DA or even discontinuing DA for ICD therapy. Observational studies were identified, but no randomised clinical trials.
3. The effectiveness of switching DA therapy to levodopa/carbidopa intestinal gel (LCIG) or levodopa/entacapone/carbidopa intestinal gel (LECIG) therapy for ICD therapy. A literature search identifies 17 relevant publications including case series and one observational study.

### Study analysis and guideline establishment

#### Effectiveness of switching between DAs for ICD therapy

Several retrospective and prospective observational studies report overall lower risk for an ICD for rotigotine compared to pramipexole or ropinirole [18, 32, 35]. The literature search did not identify any studies that evaluate the effect of switching oral DA for ICD therapy.

#### The efficacy of reducing DA doses or even omitting DAs for ICD therapy

One study reported that 80% of patients were ICD-free after DA dose reduction and 100% of patients were ICD free after DA discontinuation [36]. In a follow-up study of 30 PD patients with ICD on DA, discontinuation of DA showed a reduction in ICD severity of 50% in the first year and a further 20% in the second year [11]. Therefore, DA dose reduction and discontinuation are effective treatment strategies for ICD therapy in PD. If discontinuation is unsuccessful, for example, in the setting of DA withdrawal syndrome (DAWS), the dose should be tapered to the lowest tolerated DA dose.

#### The efficacy of switching DA therapy to a continuous levodopa/carbidopa intestinal gel for ICD therapy

Case series [37, 38] and an international prospective observational study [39] showed improvement in ICD symptoms (QUIP-RS sum score decreased by 64.4% over 6 months [39]) or “almost complete” resolution of ICD symptoms [38]. These studies included PD patients who were eligible to switch to LCIG therapy because of motor fluctuations rather than ICD symptoms alone. In conclusion, LCIG therapy has been shown to effectively treat ICD in PD patients who have an indication for continuously non-oral dopaminergic therapy.

**Table:** 

*Recommendation for the management of dopamine agonists in ICD in Parkinson’s disease (new)*
Withdrawal of dopamine agonists is an effective ICD treatment. We recommend a gradual reduction of dopamine agonist dosage In the event of adverse effects of dopamine agonist reduction (e.g. dopamine agonist withdrawal syndrome), the dopamine agonist should be reduced to the lowest tolerated dose Patients with Parkinson's disease who are eligible for continuous non-oral dopaminergic therapy may be considered for ICD therapy with levodopa-carbidopa intestinal gel (LCIG)
Level of consensus: 100%, strong consensus

### How effective are non-dopaminergic pharmacotherapies in the treatment of ICD in PD?

Although discontinuation of DA therapy is an effective ICD treatment in PD, dose reduction or discontinuation of DA may result in (i) intolerable deterioration of motor status, (ii) worsening of non-motor symptoms or (iii) a DA withdrawal syndrome. Therefore, DA therapy cannot be stopped or sufficiently reduced in every PD patient with ICD symptoms. One year after discontinuation of DA, up to 50% of PD patients still have signs and symptoms of an ICD and study reported persistent ICD in 30% of PD patients two years after DA discontinuation [11]. Therefore, further therapies such as non-dopaminergic drug therapies, e.g. drugs used in the treatment of addiction, are needed to treat ICD in PD.

### Data sources and search strategy

A literature search yielded 517 results, which were systematically searched according to the research question. There were two placebo-controlled trials and many case series and case reports.

### Study analysis and guideline establishment

Naltrexone, an opioid receptor antagonist, was evaluated in a randomised, placebo-controlled trial in 50 PD patients with an ICD. At a daily dose of 50–100 mg, naltrexone did not meet the primary endpoint (clinical global impression), but the secondary endpoint (reduction in ICD symptom severity according to QUIP-RS) was met [40]. A double-blind, crossover study investigated the efficacy of amantadine at a dose of 200 mg/d in PD patients with compulsive gambling. In this 17-week study, the dose of DA was kept constant. Amantadine abolished or significantly reduced compulsive gambling. The beneficial effect of amantadine began as early as 4 days after drug administration [41]. Unfortunately, this study only evaluated the effect of amantadine on compulsive gambling and not on ICD symptoms per se, which limits its generalisability. Case reports and one observational study describe the development of signs and symptoms of an ICD under amantadine [10, 42, 43]. In small case series zonisamide [44], clozapine [45–47], risperidone, quetiapine [47] and valproate acid [48] showed clinical benefits on the Clinical Global Impression Scale, improved results on the Barratt Impulsiveness Scale [44] or even a complete remission of ICD [45]. These drugs are not approved for use with ICD in PD.

**Table:** 

*Recommendation for the use of non-dopaminergic medications in the management of ICD in Parkinson’s disease (new)*
Non-dopaminergic drugs such as naltrexone, zonisamide, clozapine, risperidone, quetiapine and valproate acid should not be used for ICD therapy due to lack of evidence
Level of consensus: 96.7%, strong consensus

### How effective are behavioural therapies in treating impulse control disorders in PD compared with no behavioural therapy?

Often, stopping DA does not result in rapid or complete remission of ICD symptoms. In addition, treatment often leads to a worsening of the motor or non-motor condition. Therefore, the search for behavioural treatment options is another therapeutic strategy for ICD therapy in PD.

### Data sources and search strategy

The literature search yielded 11 publications as a result. After screening the abstracts and full text, further studies were extracted from the literature references and a total of 19 publications were selected for this guideline. One randomised controlled trial was found, as well as several case reports and review articles.

### Study analysis and guideline establishment

In a randomised controlled trial, the cognitive behavioural therapy included Albert Ellis' A-B-C model (Activation experiences–beliefs–consequences). 27 PD patients with an ICD were randomly assigned to a cognitive behavioural therapy group (10 patients) or a wait-list control group (17 PD patients). Cognitive behavioural therapy led to a significant improvement in ICD symptoms and scores on the Neuropsychiatric Inventory (NPI). This study demonstrated the feasibility and efficacy of cognitive behavioural therapy in the treatment of ICD in PD [49].

**Table:** 

*Recommendation for the use of cognitive behavioural therapies in the management of ICD in Parkinson’s disease (new)*
Cognitive behavioural therapy can be used to treat ICD in PD
Level of consensus: 100%, strong consensus

### How effective is STN-DBS in treating ICD in PD compared to treatment without STN-DBS in PD with an ICD?

After STN-DBS, dopaminergic medication can usually be reduced by about 50%, and motor skills are maintained or improved [50]. This reduction in medication may improve ICD in PD. Hypodopaminergic behaviour is characterised by an amotivational behaviour with low intrinsic impulsivity, clinically characterised by signs and symptoms of apathy. In contrast, a hyperdopaminergic state is characterised by high impulsivity, inventiveness and an increase in intrinsic drive to act, which together favour ICD [51]. Fluctuations between these two states may be experienced as increased non-motor fluctuations due to the ups and downs of pulsatile dopaminergic stimulation. After STN-DBS, these non-motor fluctuations decrease and the level of dopaminergic stimulation remains more constant [52]. The reduction in dopaminergic medication associated with STN-DBS is accompanied by a desensitisation of dopaminergic receptors [53]. A desensitising effect is thought to be at the core of the beneficial effect of STN-DBS on the ICD [54]. However, STN-DBS can also increase impulsivity [55] and in some cases trigger an ICD.

### Data sources and search strategy

A literature search identified 92 results, mostly case reports, case series, retrospective observations and one prospective study as well as numerous reviews and expert opinions. Studies that report incomplete data (five studies [56–60]) included “a spectrum of impulse control and related disorders” [61] (1 study) or report unilateral DBS (1 study) were excluded from further analysis.

### Study analysis and guideline establishment

A subanalysis from the Earlystim trial reported a reduction in non-motor fluctuations and a reduction in hyperdopaminergic behaviour after STN-DBS [54]. Another retrospective study of 69 PD patients showed a significant reduction in hyperdopaminergic behaviour as measured by the Ardouin scale [62]. A case series of 7 PD patients with gambling addiction reported complete remission 18 months after STN-DBS [63]. Other case series with a total of 5 PD patients report a positive course of ICD symptoms after STN-DBS [58, 64, 65]. In a recent prospective study, 217 PD patients were treated with STN-DBS. 22 out of 23 patients with an ICD showed complete remission ICD signs and symptoms 1 year after surgery. However, eight patients developed a new ICD after STN-DBS [66].

STN-DBS can lead to partial or complete remission of ICD in PD by reducing dopaminergic medication [58, 62–65]. However, the interplay between medication reduction and stimulation-induced increase in impulsivity is complex and cannot be predicted for every patient with ICD. Reduction of dopaminergic medication after surgery may lead to apathy [62, 67], which needs to be considered in postoperative management. There are no studies investigating the effects of STN-DBS on ICD in PD beyond the established indications for STN-DBS. This limits the strength of the recommendation given in this guideline.

**Table:** 

*Recommendation for the use of deep brain stimulation in the management of ICD in Parkinson’s disease (new)*
Bilateral deep brain stimulation of the subthalamic nucleus can be recommended to treat signs and symptoms of an ICD in patients eligible for deep brain stimulation
Level of consensus: 100%, strong consensus

### Psychosis

Psychosis associated with PD, often characterised by hallucinations and delusions, is a complex challenge that affects an estimated 30–40% of PD patients at some point during their illness [68, 69]. Psychosis in PD can manifest with a wide range of symptoms, ranging from mild, non-bothering hallucinations to severe, distressing delusions that significantly impair daily functioning. Psychosis in PD not only reduces the quality of life but also complicates treatment strategies aimed at managing the motor symptoms of the disease [68–72]. The psychophysiology of psychosis in PD is complex, involving dopaminergic, cholinergic and serotonergic pathways, making its management particularly challenging. Treatment recommendations emphasise a nuanced approach, prioritising patient safety and quality of life. The most important obstacle in treating psychosis in PD is that most available antipsychotics impact motor function. First steps include reviewing and adjusting medications that may exacerbate psychotic symptoms, particularly dopaminergic therapy, and identifying other aggravating factors that can cause secondary psychosis. If pharmacological interventions for psychosis are required, antipsychotic medications are considered, with a preference given to those with a lower risk of worsening motor symptoms, such as clozapine and quetiapine, although they require careful monitoring due to potential substance-specific side effects [68–72].

### What is the nature and symptoms of psychosis in PD?

The prodromal phase typically includes vivid dreams and nightmares in most cases. This phase may be followed by the development of illusions (e.g. misidentifying objects as people) and a false sense of the presence of objects or beings. Patients then typically develop pseudohallucinations with preserved insight. For example, a patient may see a family pet that died years ago, or insects crawling on the walls. The content of these pseudohallucinations is usually recurrent. Finally, patients may develop hallucinations without insight and/or delusions [68, 70–72].

### Data sources and search strategy

The evidence is based on several reviews and case studies [68, 70–72]

### Study analysis and guideline establishment

The spectrum of psychotic disorders includes hallucinations with loss of insight and the development of delusions, e.g. delusions of jealousy, and should be carefully considered during the history and examination as these symptoms are often not spontaneously reported [68, 70–72]. An important differential diagnosis is psychosis due to delirium on PD.

**Table:** 

*Recommendation for screening for signs and symptoms of psychosis in the medical history of patients with Parkinson's disease (new)*
Psychotic and prodromal symptoms, such as vivid dreams and nightmares, misidentification of objects as persons, a false sense of the presence of objects or beings, pseudohallucinations with preserved insight (often initially of insects or pets), hallucinations and/or delusions, should be regularly assessed in people with PD throughout the course of the disease. Family members should be involved in this process
Level of consensus: 100%, strong consensus

### What are the risk factors for psychotic disorders in PD?

#### Data sources and search strategy

No controlled studies were identified that prospectively investigated risk factors for psychotic disorders in PD. Six reviews and three case studies were considered relevant [68–70, 72–77].

#### Study analysis and guideline establishment

The spectrum of risk factors is wide. Knowledge of risk factors for psychotic disorders in PD should be taken into account in preventive measures and treatment. Mild cognitive impairment or dementia, depression, advanced age, duration and severity of PD, as well as significant psychiatric (especially current or past addiction) or medical comorbidities (e.g. pronounced cerebral microangiopathy) are risk factors for the development of psychosis in PD. Other important and modifiable risk factors include sensory deprivation due to reduced vision or hearing. In addition, there are significant pharmacological risks, such as complex medication regimens (differential diagnosis with delirium). Visual hallucinations are most common in poor lighting conditions (e.g. poorly lit wards, lack of contrast in room design).

**Table:** 

*Recommendation to pay attention to the following risk factors for psychosis in Parkinson’s disease (new)*
The constellation of risk factors for psychosis in Parkinson's disease is extensive and requires vigilant attention during the patient's medical history and clinical assessment. Clinicians should ensure thorough identification and assessment of potential risk factors, including cognitive dysfunction, depressive symptoms, senescence, chronicity and severity of PD, coexisting psychiatric or somatic comorbidities, sensory deficits (particularly for uncorrected vision and hearing) and the complications of polypharmacy, during the patient history taking process
Level of consensus: 100%, strong consensus

### What diagnostic tools are available for psychotic disorders in PD?

#### Data sources

One controlled study was identified that prospectively evaluated a diagnostic tool for psychotic disorders in PD [78]. The literature search identified six review articles as relevant [68–70, 72–74].

#### Study analysis and guideline establishment

There is currently no validated diagnostic tool exists for the identification of psychotic disorders in PD. In 2022, Greger et al. developed an instrument for the targeted detection of psychotic disorder symptoms in PD [78]. In 2007, a working group from the National Institute of Neurological Disorders and Stroke (NINDS) and the National Institute of Mental Health (NIMH) developed a set of diagnostic criteria for psychotic disorders in PD. These are based on a diagnosis of PD according to the UK Brain Bank criteria for PD, at least one characteristic symptom of psychosis, including illusions, paranoia, hallucinations or delusions, and the onset of symptoms after the diagnosis of PD [79]. There are several other scales, such as the Neuropsychiatric Inventory (NPI) for patients in advanced stages, the Schedule for Assessment of Positive Symptoms (SAPS), the Positive and Negative Syndrome Scale (PANSS) or the Brief Psychiatric Rating Scale (BPRS). In 2008, the Movement Disorder Society evaluated several scales for diagnosing psychotic disorders in PD and concluded that no single scale was adequate. They recommended using a combination of different scales depending on the patient's condition [80].

**Table:** 

*Recommendation to screen for psychosis in Parkinson’s disease (new)*
Several diagnostic tools (questionnaires) are available. Accordingly, a tool for the diagnosis of psychotic disorders should be used, particularly when there is diagnostic uncertainty in PD. The Movement Disorder Society recommends that the choice of tool should be case specific. However, it is noted that training is required to become proficient in the use of these scales. The NINDS-NIMH diagnostic criteria should be incorporated into the diagnostic process for psychotic symptoms in people with PD as an adjunct to clinical assessment
Level of consensus: 100%, strong consensus

### How are psychotic symptoms diagnosed in PD?

#### Data sources and search strategy

No controlled studies were identified that prospectively evaluated a diagnostic tool for psychotic disorders in PD. The literature search identified six review articles as relevant [68–70, 72–74].

#### Study analysis and guideline establishment

There is no standardised methodology for the diagnosis of psychotic symptoms in PD. Before diagnosing PD medication-induced psychosis as the cause of a psychotic syndrome, other aetiologies causing secondary psychosis must be excluded. Important differential diagnoses that must be considered include other neurodegenerative disorders, psychosis associated with the various causes of delirium, major depressive disorder, side effects of dopaminergic medications (especially dopamine agonists, COMT inhibitors), side effects of other medications (e.g. opiates, anticholinergics), hyponatremia, substance abuse or intoxication. To date no standardised tool has been developed for the diagnosis of psychotic disorders in PD. Various questionnaires may facilitate the diagnostic process (see research question above).

**Table:** 

*Recommendation to diagnose psychosis in Parkinson’s disease (new)*
Several diagnostic tools have been developed (see recommendation for screening for psychosis). Psychotic symptoms in people with PD should be diagnosed primarily clinically, taking into account risk factors and historical data. In cases of diagnostic uncertainty, several rating scales (see recommendations for screening for psychosis) can be used. A thorough exclusion of differential diagnoses should be carried out
Level of consensus: 96.8%, strong consensus

### Do preventive measures reduce the risk of psychosis in people with PD?

#### Data sources

No controlled trials were identified that prospectively evaluated preventive measures in people with PD to reduce the risk of psychosis. The literature search identified five review articles as relevant [68–70, 72, 81].

#### Study analysis and guideline establishment

Given the environmental factors that increase the risk of psychosis in PD, the question arises as to whether preventive measures can reduce the likelihood of its manifestation. General strategies to reduce the risk of psychosis should be used in the management of all patients with PD. These strategies include maintaining circadian rhythms, optimising normal sensory input (e.g. use of auditory and visual aids, adequate lighting) or maintaining a family environment. Specific comorbidities such as infection and dehydration should also be addressed. The use of unnecessary medications, especially those with anticholinergic, antiglutamatergic or sedative properties, should be avoided. Polypharmacy should be reduced as much as possible.

**Table:** 

*Recommendation to prevent psychosis in Parkinson’s disease (new)*
Preventive measures such as maintaining the circadian rhythm, normalising sensory inputs, maintaining a familiar environment, treating specific medical comorbidities (e.g. infections, dehydration, etc.), correcting visual and hearing impairments and avoiding unnecessary medications (especially anticholinergic, antiglutamatergic, or sedative medications) should be used in PD patients
Level of consensus: 100%, strong consensus

In addition to that recommendation, the guideline recommends to follow the other recommendation of this chapter (especially improvement of modifiable risk factors, medication managements) to avoid the onset of psychotic symptoms.

### Does a change in Parkinson's disease therapy in people with PD who experience psychosis lead to a reduction in psychotic symptoms?

#### Data sources

No controlled trials were identified that prospectively investigated the effects of changing anti-Parkinson’s medications in people with PD and psychotic symptoms in terms of effectiveness in reducing psychotic experiences. Five review articles were considered relevant in the literature search [68–70, 72, 81].

#### Study analysis and guideline establishment

Given that anti-Parkinson’s medications can induce psychotic symptoms in people with PD, it stands to reason that a change in PD therapy may lead to a reduction in psychotic symptoms. The dose of anti-Parkinson’s medication can be reduced as much as the movement disorder symptoms allow. The regimen should be changed to a simplified strategy of avoiding anticholinergics first, followed by a stepwise reduction of amantadine, MAO-B inhibitors or dopamine agonists, in that order. In addition, it may be beneficial to avoid COMT inhibitors and extended release formulations of dopaminergic medications.

**Table:** 

*Recommendation to change anti-parkinsonian medication in the management of psychosis in Parkinson’s disease (new)*
In the event of psychotic symptoms in people with PD, medication should be adjusted as follows: 1) Dose reduction of anti-parkinsonian medication 2) Simplification of combination treatments 3) Reduction/discontinuation should follow this sequence: Anticholinergics > Amantadine > MAO-B inhibitors > Dopamine agonists > COMT inhibitors
Level of consensus: 100%, strong consensus

### Does treatment with acetylcholinesterase inhibitors in individuals with PD experiencing psychosis lead to a reduction in psychotic symptoms?

#### Data sources

No completed controlled trials were identified that prospectively evaluated treatment with acetylcholinesterase inhibitors (AchE inhibitors) in people with PD with psychotic symptoms for efficacy in reducing psychotic experiences. One prematurely terminated trial was identified [82]. The literature search identified five review articles as relevant [68–70, 72, 81].

#### Study analysis and guideline establishment

AchE inhibitors may reduce the symptoms of cognitive deficits. As cognitive deficits are a risk factor for the development of psychosis in PD, it is conceivable that treatment with AchE inhibitors could lead to a reduction of psychotic experiences. The limited data from a discontinued trial investigating whether treatment with an AchE inhibitor in PD patients with mild visual hallucinations delays progression to psychosis suggest a watchful waiting approach rather than early treatment with rivastigmine. Several review articles recommend acetylcholinesterase inhibitors for severe visual hallucinations.

**Table:** 

*Recommendation in the management of psychosis in Parkinson’s disease, the use of acetylcholinesterase inhibitors (new)*
As a result of the limited evidence, a therapeutic trial of AchE inhibitors may be considered in people with PD and psychosis when other measures fail. This is an off-label use. Potential interactions at the level of CYP enzymes as well as cardiac and gastrointestinal side effects should be carefully considered
Level of consensus: 100%, strong consensus

### Does antipsychotic treatment reduce psychotic experiences in people with PD?

#### Data sources

The literature search identified different sources of evidence (RCTs, systematic reviews, meta-analyses). After the search was completed, a comprehensive meta-analysis was published [83], which was used as the basis for the recommendation. This meta-analysis is methodologically robust and has a level of highest evidence. This network meta-analysis included 19 studies with 1242 participants with PD psychosis and investigated pimavanserin, quetiapine, olanzapine, clozapine, ziprasidone and risperidone. In this meta-analysis, only clozapine and pimavanserin showed sufficient suppression of psychotic symptoms without worsening motor function. Quetiapine was found to worsen cognition.

#### Study analysis and guideline establishment

Given that antipsychotics can reduce psychotic experiences in psychoses of different origins, it seems plausible that they could also reduce psychotic experiences in the treatment of people with PD and psychosis. However, the mechanism of action of most antipsychotics, primarily through D2 receptor blockade, precludes their use in PD. Severe psychotic symptoms associated with PD require specific antipsychotic pharmacotherapy when no better explanation is found after consideration of differential diagnoses, when adjustment of anti-parkinsonian medication does not improve psychotic experiences and when non-pharmacological treatment does not eliminate or control existing symptoms. Of the approved treatments for psychotic disorders, only clozapine, quetiapine and olanzapine have no relevant effect on motor function. Olanzapine should not be offered in PD because of its significant anticholinergic properties. Based on the cited meta-analysis, clozapine should be offered for the treatment of psychosis in PD. Clozapine has been shown to be effective in treating psychosis in PD at doses of 6.25–50 mg per day. However, due to the risk of agranulocytosis and myocarditis and the consequent need for regular monitoring, together with other side effects such as sedation, sialorrhoea (risk of aspiration, increased risk of pneumonia), slowed gastrointestinal transition (risk of constipation or even ileus) and risk of falls as a consequence of hypotension [84], it is not always the first choice of treatment. Instead, quetiapine is often used in clinical practice because it requires less monitoring and has a lower risk of extrapyramidal side effects. However, the meta-analysis showed no efficacy of quetiapine at doses of 25–150 mg per day in the treatment of psychosis in PD, and dyscognitive effects were observed. Quetiapine may have anticholinergic properties in higher doses due its metabolite norquetiapine.

The dilemma of balancing efficacy and safety in the treatment of PD psychosis with clozapine or quetiapine remains, but may be resolved in the future with pimavanserin [83, 85]. However, pimavanserin is not currently approved in Europe. The high daily treatment cost remains a limiting factor for its use. A meta-analysis based on 14 studies (1 RCT, 9 prospective observational studies, 4 retrospective studies) showed that electroconvulsive treatment improves psychotic, other non-motor and motor symptoms in people with PD [86]. Benzodiazepines with a short half-life may be considered from a clinical-pragmatic point of view, but there is no randomised trial evidence and the increased risk of falls, daily sleepiness, negative impact on cognition and potential paradoxical reactions limit their use (expert opinion).

**Table:** 

*Recommendation in the management of psychosis in Parkinson’s disease, the use of antipsychotic drug therapy (updated)*
Clozapine should be offered to people with Parkinson's disease and psychotic symptoms when other strategies (adjustment of anti-parkinsonian medication; non-pharmacological measures) fail, with the necessary education about the possible side effects of agranulocytosis and myocarditis, and with the necessary monitoring
Level of consensus: 100%, strong consensus
Quetiapine may be used to treat psychosis in PD patients without pre-existing cognitive impairment. Patients should be informed about the low evidence level for its efficacy
Level of consensus: 92.9%, strong consensus
Olanzapine and other antipsychotics not explicitly recommended in this guideline should not be offered to individuals with PD and psychotic symptoms
Level of consensus: 100%, strong consensus
In PD patients with psychotic symptoms, a stepwise approach should be preferred: 1. Implementation of general non-pharmacological measures (including sensory shielding, reorientation measures, restoration of circadian rhythm) 2. Implementation of general therapeutic measures (including treatment of dehydration, infection, etc.) 3. Reduction/adjustment of triggering drugs in general (anticholinergic, antiglutamatergic, sedative) and anti-Parkinson's drugs (especially amantadine, MAO-B inhibitors, dopamine agonists and COMT inhibitors, combination treatments) 4. Clozapine should be offered after failure of steps 1–3, after appropriate risk–benefit analysis (risk of agranulocytosis, risk of myocarditis, risk of falls, anticholinergic side effects). Alternatively, quetiapine may be offered off-label to people with PD without cognitive impairment 5. In the case of cognitive impairment and failure of steps 1–3, a switch to an acetylcholinesterase inhibitor may be offered 6. If steps 1–4 and possibly step 5 fail, electroconvulsive therapy may be offered
Level of consensus: 100%, strong consensus

### Delirium

PD is an independent risk factor for the development of delirium. The development of delirium is an important complication that is associated with a high risk of long-lasting deterioration of motor, autonomic and higher brain functions and psychopathology. Delirium is also associated with higher mortality rates. The few studies available do not allow a statement to be made as to whether delirium in the context of PD is characterised by a specific phenotype. During delirium, an increase in motor symptoms and a reduced response to dopaminergic medication may occur. The clinical diagnosis of delirium in PD requires the differentiation of symptoms inherent to PD and delirious symptoms. This includes both motor symptoms such as hypokinesia and the various non-motor symptoms that can occur in the different stages of PD. Neuropsychiatric symptoms, not only hallucinations and delusions but also apathy, restlessness, insomnia and sleep-associated behavioural disorders, overlap with the possible symptoms of delirium. Behavioural disorders and fluctuations in vigilance are particularly common in dementia with Lewy bodies (DLB). In addition, side effects of anti-Parkinson's therapy can lead to symptom overlaps. These include psychomotor agitation, visual hallucinations, vegetative symptoms, agitation and confusion. To date, there is insufficient knowledge about the prevalence, incidence, course and prognosis of delirium in PD. There is also a lack of clinical studies from which evidence-based recommendations for the management of delirium in PD can be drawn.

In sum, the overlap between symptoms of delirium and PD and the complex role of dopaminergic transmission for phenotype and pharmacotherapy motivated us to define guidelines for delirium in PD in addition to the established guidelines for delirium.

### What are the risk factors for delirium in PD?

#### Data sources, study analysis and guideline establishment

No controlled studies were identified in which specific risk factors for delirium in PD were investigated. General risk factors for the development of delirium [87] were identified in two reviews and judged to be also relevant for delirium in PD [88, 89].

**Table:** 

*Recommendation to pay attention to the following risk factors for delirium in Parkinson’s disease (new)*
• The following factors can predispose to the occurrence of delirium: Advanced age (usually > 65 years), pre-existing substance use, polypharmacy, hearing impairment, internal comorbidities and cognitive disorders. Infections, metabolic disorders, psychological stressors, drug side effects and pain can be considered as triggers. Prolonged treatment in hospital, especially in intensive care units, major surgical interventions and catheters, can increase the risk of delirium in hospitalised patients
Level of consensus: 100%, strong consensus

### What diagnostic tools exist for delirium in PD?

#### Data sources, study analysis and guideline establishment

No studies were identified in which diagnostic instruments for delirium in PD were investigated. According to three reviews, it is possible to use scales that are not specifically developed for the diagnosis of delirium in PD [88–90].

**Table:** 

*Recommendation on screening methods for the detection of delirium in Parkinson's disease (new)*
For delirium screening, the Nurse Delirium Screening Scale (Nu-Desc) [91] and the Delirium Observation Scale (DOS-S) [92] may be used. The Confusion Assessment Method (CAM) [87] may be used to diagnose delirium and assess its course
Level of consensus: 100%, strong consensus

### How is delirium diagnosed in PD?

#### Data sources and search strategy

No controlled studies were identified in which specific diagnostic criteria for delirium in PD were investigated. Three reviews were identified that addressed the diagnosis of delirium in PD [88–90].

#### Study analysis and guideline establishment

In principle, the criteria listed in ICD-10/11 and DSM-V apply to the diagnosis of delirium. According to these, delirium is defined as an acute and transient disturbance of attention and perception with accompanying disturbance of memory, orientation, language and perception. In ICD-11 and DSM-V, delirium is additionally characterised by a fluctuating course. Disturbances of the sleep–wake rhythm, hallucinations and delusions and psychomotor agitation are further symptoms that can be associated with delirium.

Delirium presents in three clinical manifestations: (I) the hyperactive, (II) the hypoactive form and (III) mixed delirium, which is characterised by alternating hyperactive and hypoactive states. The mixed form is the most common. The diagnosis of delirium in PD is complicated by overlapping symptoms of delirium with those that can be attributed to PD symptoms of delirium with those of PD (e.g. apathy, vigilance fluctuations, drug-induced hallucinations). Imaging, EEG and laboratory tests are performed for differential diagnosis and identification of triggering factors.

**Table:** 

*Recommendation for the diagnosis of delirium in Parkinson's disease (new)*
The diagnosis of delirium in PD can be made on the basis of the criteria listed in ICD-10/11 or DSM-V criteria listed in the ICD-10/11 or DSM-V. Possible overlaps with other psychopathological complications of PD should be taken into account. The Confusion Assessment Method (CAM, [1]) may be used to diagnose delirium in PD. Differential diagnoses and triggering factors for delirium can be identified by paraclinical diagnostics
Level of consensus: 100%, strong consensus

### Do preventive measures in PD lead to a reduction in the risk of delirium?

#### Data sources and search strategy

No controlled studies were identified in which preventive measures to avoid delirium in PD were investigated. Two reviews were identified that dealt with the prevention of delirium in PD [88, 89].

#### Study analysis and guideline establishment

Prevention includes adherence to the day–night rhythm, use of assistive devices, early mobilisation, adequate food and fluid intake, a quiet environment and, as far as possible, avoidance of catheters and accesses [93, 94]. Adequate hydration and the treatment of pain and infections are relevant both for the prevention as well as for the treatment of delirium. Training hospital staff on delirium is desirable. The involvement of relatives plays a crucial role in delirium prevention [87]. If possible, the admission of an accompanying person can lower the risk of hospitalisation delirium in particularly vulnerable patients, e.g. those with dementia or psychotic symptoms [88, 89].

**Table:** 

*Recommendation for the prevention of delirium in Parkinson's disease (new)*
The following measures may be considered to prevent delirium in patients at risk of delirium: 1. early mobilisation 2. avoidance of catheters and i.v. lines 3. adherence to the day–night rhythm 4. sensory aids (hearing aids, visual aids) 5. food and fluid intake 6. quiet environment 7. environment with good lighting and good colour contrasts 8. treatment of pain and infections 9. presence of caregivers (including rooming-in) 10. Reorientation measures (e.g. introducing oneself by name at every contact, clock in the in the field of vision)
Level of consensus: 96.9%, strong consensus

### Does a change in Parkinson's therapy in PD lead to a reduction in delirium?

#### Data sources and search strategy

No controlled studies were identified in which the effects of a change in PD therapy on delirium were investigated. Three reviews were identified that addressed the effects of PD therapy on delirium in PD [88, 89, 95].

#### Study analysis and guideline establishment

In cases of delirium, drugs with potentially higher delirogenic potency should be discontinued [96]. These include DA, anticholinergics and amantadine. This often results in levodopa monotherapy [88]. Close monitoring is required since withdrawal symptoms (DWAS: dopamine agonist withdrawal syndrome; MDES: malignant dopaminergic withdrawal syndrome) can occur after abrupt discontinuation of these substances.

**Table:** 

*Recommendation for changing the anti-Parkinson medication for delirium in Parkinson's disease (new)*
In the case of delirium in PD, amantadine, anticholinergics and dopamine agonists should be reduced or (gradually) be discontinued
Level of consensus: 93.1%, strong consensus

### Do other pharmacological therapies lead to a reduction in delirium in PD?

#### Data sources and search strategy

No controlled trials were identified in which the effects of other pharmacological therapies on delirium in PD were investigated. Three reviews were identified that dealt with the pharmacological treatment of delirium in PD [88, 89, 95].

#### Study analysis and guideline establishment

Drug therapy options include the treatment of factors potentially causing delirium, such as acute infections, pain or metabolic disorders. Antipsychotic treatment of delirium with quetiapine and clozapine has not yet been sufficiently investigated in PD, while other antipsychotics are contraindicated for the treatment of delirium in PD due to their dopamine blocking properties. Due to its anticholinergic properties, clozapine can increase delirium. Quetiapine is used in other indications (e.g. delirium in dementia), but corresponding studies are lacking, as is the use of benzodiazepines for severe anxiety and agitation. The administration of benzodiazepines increases the risk of falls and aspiration and must therefore be used with extreme caution. Paradoxical effects are also possible.

**Table:** 

*Recommendation for drug therapy of delirium in Parkinson's disease (new)*
In the case of delirium in PD, triggering factors (pain, infections, metabolic disorders) should be treated with medication. Quetiapine may be used to treat delirium. When using quetiapine, cognition, blood pressure and bladder emptying should be monitored. It must be noted that Quetiapine has not been specifically investigated for delirium in PD [97]. Clozapine and benzodiazepines may be used in individual cases with special consideration of possible side effects, especially the risk of falls. In general, due to the anticholinergic properties, a worsening of delirium in PD can also occur with the antipsychotics mentioned here
Level of consensus: 93.1%, strong consensus

### Do non-pharmacological therapies in PD lead to a reduction in delirium?

#### Data sources and search strategy

No controlled trials were identified in which non-pharmacological interventions were used to reduce delirium in PD. Three reviews were identified that dealt with non-pharmacological measures to reduce delirium in PD [88, 89, 95].

#### Study analysis and guideline establishment

Non-pharmacological measures play a decisive role both for prevention of delirium in patients at risk and in manifest delirium (see above). Involving relatives supports the communication and (re)orientation of those affected by delirium [94]. If possible, it is helpful if the patient's partner or a close caregiver is also admitted to the hospital [89]. Adapted ward routines with direct nursing care and therapeutic measures for orientation during the day and at night have a positive effect on patients with delirium. An adequate supply of food and fluids and the provision of hearing aids are further important elements of non-medication therapy [87].

In the case of delirium in PD, triggering factors (pain, infections, metabolic disorders) should be treated with medication. Quetiapine can be used to treat delirium under control of cognition, circulation and bladder emptying. It must be noted that Quetiapine has not been specifically investigated for delirium in PD [97]. Clozapine and benzodiazepines can be used in individual cases with special consideration of possible side effects, especially the risk of falls. In general, due to the anticholinergic properties, a worsening of delirium in PK can also occur with the antipsychotics mentioned here.

**Table:** 

*Recommendation for the non-drug therapy of delirium in Parkinson's disease (new)*
The following measures may be used for the non-medication therapy of delirium in PD: 1. early mobilisation 2. avoidance of catheters and accesses 3. adherence to the day–night rhythm 4. sensory aids (hearing aids, visual aids) 5. food and fluid intake 6. quiet environment 7. environment with good lighting and good colour contrasts 8. treatment of pain and infections 9. presence of caregivers (including rooming-in) 10. reorientation measures (e.g. introduction by name at every contact, clock in the field of vision)
Level of consensus: 97.0%, strong consensus

## Discussion

This guideline article presents evidence for and expert opinions on diagnostic and therapeutic procedures in the management of ICD, psychosis and delirium in PD. It is acknowledged that only a few randomised controlled studies in these fields of interest have been performed. In the treatment of those neuropsychiatric conditions, it is important to consider the individual characteristics of patients. These differences between patients, such as demographic factors, their comorbidities, the cognitive profile and cognitive reserve, and individual disease characteristics and differences in the course of the disease, need to be considered in both diagnosis and treatment regimes. In the following sections, we will discuss some of the special considerations that should be taken into account. We will also describe some of the medical conditions that may occasionally require a deviation from the guidelines, and we will suggest ways to address some of the gaps in knowledge that could be beneficial for everyday clinical practice.

The consequences of an ICD can be serious for patients, and their management can be challenging. It is therefore important to assess individual risk factors. Diagnosing an ICD can be challenging, especially if the person does not feel any subjective distress or derives short-term comfort and satisfaction from the ICDs, or hide its symptoms if stopping it leads to dysphoria and anxiety. Regular screening for an ICD and education of patients, family members and caregivers remain essential. One study reported a high proportion of subsyndromal ICDs [98]. It would be beneficial to develop and validate measures that can detect even small changes in impulsivity during DA treatment that may indicate a tendency towards ICD, and thus stratify patients’ risk of developing an ICD. First line treatment focuses on the gradual reduction of DAs and replacing DAs with fractionated levodopa doses. Given the differences in DA on dopamine receptor sub-type affinity, changing the DA might be a therapeutic strategy. Studies comparing the ICD risk of different DAs head to head are lacking. It is important to consider the possibility of a dopamine agonist withdrawal syndrome (DAWS) lowering the dosage of DAs or stopping DA medication. DAWS can occur in up to 19% of cases [99]. Additionally, the development of an apathy must be taken into account. By tapering the DAs slowly, it is less likely that both behavioural reactions to DA reduction will be observed. It is a challenge to find a balance between the DA reduction, consecutive effects on ICD symptomatology and undesirable effects of medication reduction in the motor and the non-motor domain. In the area of ICD treatment, only one RCT demonstrated a positive result, that is, a specific psychotherapy that has been shown to be effective in treating ICD in PD patients, although its regional availability may be limited.

It would appear that studies investigating the impact of non-oral continuous drug delivery and deep brain stimulation on ICD have indicated positive effects on ICD in the majority of cases. In the case of STN-DBS, this may be related to the associated reduction in medication. However, it is also worth considering the possibility of a specific stimulation effect. It has been observed that more ventral stimulation can provoke increased affective and especially (sub-) manic behaviour, which can be reversed by switching to dorsal (the higher localised) stimulation contacts [100]. While levodopa-carbidopa intestinal gel infusion does not necessarily result in a reduction of the daily levodopa equivalent dose, it does achieve a more constant levodopa plasma concentration, which result in a less pulsatile effect of dopamine. It is also worth considering the possibility that foslevodopa, which is less invasive because it is administered subcutaneously, might also be a candidate to treat signs and symptoms of ICD. Further studies are needed to confirm this hypothesis.

The treatment of psychotic symptoms in PD remains challenging. In principle more than 30 different antipsychotics are available that were shown to be effective in improving psychotic symptoms in general [101]. However, it is evident that most antipsychotics cannot be offered in PD due to the risk of significant motor side effects. From the currently available antipsychotics, only quetiapine, olanzapine and clozapine have a tolerable motor side effect profile. However, due to its anticholinergic properties olanzapine is not recommended in the guideline anymore. In clinical practice the dilemma that quetiapine is more easy to use and has a more favourable side effect profile than clozapine, but that clozapine has the more convincing evidence profile becomes clear. In the real-world situation this dilemma must be discussed on an individual case-by-case basis and we decided to priorities clozapine, but to add quetiapine as an alternative in PD patients with psychosis and no relevant cognitive impairments. Moreover, the inverse agonist serotonin 5-HT2A receptor pimavanserin was introduced in 2017 as an alternative option for psychosis in PD. The guideline acknowledges the potential of pimavanserin, but due to the unavailability Europe, no specific recommendation was made. Pimavanserin has been shown to be effective in reducing psychotic symptoms in PD without any relevant motor side effects [83]. The most important side effect is a potential QTc prolongation. Whether pimavanserin would be as effective as, e.g. clozapine remains elusive and as no head-to-head studies will be performed by the manufacturer, we will have to wait future results of network meta-analysis. Breakthroughs in the field of antipsychotic treatment are sparse. However, several non-dopaminergic antipsychotics have been introduced and tested in Phase II and III trials within the last three years. Examples are Trace amine-associated receptor 1 agonists (Phase III trials failed), D-amino acid oxidase inhibitors (currently in Phase II) and cholinergic antipsychotics. The latter are at this stage the most promising compounds. Here, xanomeline–trospium chloride is the most promising agent. Xanomeline is an agonist at M1/M4 muscarinic receptors and this is combined the peripheral muscarinic receptor antagonist trospium chloride to reduce cholinergic side effects. This combination was successful in Phase II and III schizophrenia trials [102, 103] and may also be promising for psychotics in PD as it has no antidopaminergic properties. Another potential compound from this group is emraclidine, an allosteric modulator at M4 receptors. This drug is in an early-stage clinical trial programme [104], but is considered to be another promising option in the development of non-dopaminergic antipsychotics. There is light at the end of the tunnel regarding new non-dopaminergic antipsychotics for the treatment of schizophrenia and if successful those compounds will also be tested in PD psychosis. However, in the end the best clinical option to do the best we can to prevent the onset of psychosis in PD.

At present, there are only few studies addressing delirium in PD. Given that delirium in PD is a potentially avoidable and treatable condition, more efforts are needed to improve recognition and to improve prevention and management. Acute onset may help differentiate between delirium and chronic neuropsychiatric symptoms inherent to PD. Yet, the clinical course may include fluctuations of symptoms in both conditions and delirium can thus be easily overlooked in PD. Psychomotor slowing in hypoactive delirium may be particularly difficult to differentiate from hypodopaminergic OFF-state conditions. Available assessment tools for delirium, such as CAM [87], should be validated for PD or modified accordingly. Further research is also needed to better understand transient or permanent motor deterioration which often occurs in the context of delirium in PD and may not properly respond to levodopa medication. At present, the knowledge about the effects of pharmacologic interventions on the course of delirium in PD is insufficient and pertinent recommendations largely rely on expert opinion. Prospective multicentre approaches are needed to delineate risk factors, prevalence, management and outcome of delirium in PD.

Delirium is a burdensome situation also for caregivers and relatives. More insight on caregiver burden and support is critical and may be relevant to reduce long-term institutionalisation following delirium. In this line, awareness for PD delirium should be increased by continuous education of physicians, nursing staff and caregivers. More efforts and appropriate reimbursement are needed to implement preventive and supportive measures such as screening routines to identify PD patients at risk for delirium, rooming-in of caregivers, psychosocial counselling and cognitive rehabilitation.

## Data Availability

Systematic literature search data will be shared by request from any qualified investigator. Data sharing requests are made in writing through Prof. Dr. Karsten Witt (chapter “ICD”, karsten.witt@uni-oldenburg.de, Carl von Ossietzky Universität, Oldenburg), Prof. Dr. Johannes Levin (chapter “Psychosis”, johannes.levin@med.uni-muenchen.de, University Munich) and Prof. Dr. Georg Ebersbach (chapter “Delirium”, ebersbach@kliniken-beelitz.de, Movement Disorder Hospital, Beelitz-Heilstätten). Data sharing requires a formal data sharing agreement with approval from the respective University and the guideline office of the German Society of Neurology (DGN). Data sharing agreements must include details on how the data will be stored, who will have access to the data and intended use of the data and agreements as to the allocation of intellectual property.
